# Crystal structure of poly[{μ_3_-(*E*)-3-[3-(carboxyl­atometh­oxy)phen­yl]acrylato-κ^3^ *O*,*O*′:*O*′′:*O*′′′}[μ_2_-3-(pyridin-4-yl)-1*H*-pyrazole-κ^2^ *N*:*N*′]cobalt(II)]

**DOI:** 10.1107/S205698901601402X

**Published:** 2016-09-09

**Authors:** Can Zhao, Xiao-Zong Li, Hong-Lan Kang, Yi-Hang Wen

**Affiliations:** a Zhejiang Key Laboratory for Reactive Chemistry on Solid Surfaces, Institute of Physical Chemistry, Zhejiang Normal University, Jinhua, Zhejiang 321004, People’s Republic of China

**Keywords:** crystal structure, metal–organic coordination compounds, two-dimensional polymeric structure, one-dimensional helical chain, (*E*)-3-[3-(carb­oxy­meth­oxy)phen­yl]acrylic acid

## Abstract

The title compound, [Co(C_11_H_8_O_5_)(C_8_H_7_N_3_)]_*n*_, which is based on (*E*)-3-[3-(carb­oxy­meth­oxy)phen­yl]acrylic acid (H_2_ *L*) and 3-(pyridin-4-yl)pyrazole (pp) ligands, has been synthesized under solvothermal conditions. The dihedral angle between pyrazole and pyridine rings in the pp ligands is 23.1 (2)°. In the crystal, helical chains formed by pp and *L* ligands connected to the Co^II^ atom propagate parallel to the *c* axis. Co^II^ atoms of adjacent chains are bridged by the acrylic acid groups of *L* ligands into corrugated polymeric sheets in the *ac* plane.

## Abstract 2

A two-dimensional polymeric structure based on (*E*)-3-(3-(carb­oxy­meth­oxy)phen­yl)acrylic acid (H_2_ *L*) and 3-(pyridin-4-yl)pyrazole (pp) ligands, has been successfully synthesized under solvothermal conditions. In the crystal, helical chains formed by pp and *L* ligands connected to the Co metal, propagate parallel to the *a* axis.

## Chemical context   

The rational design and synthesis of metal–organic frameworks (MOFs) with multi-carboxyl­ate ligands and metal atoms has attracted much attention in coordination chemistry due to the varied topologies and potential applications in catalysis, gas adsorption, photochemistry *etc* (Fernández *et al.*, 2016 ▸). The versatility of metal–organic chemistry offers the opportunity to construct multifunctional materials based on the assembly of mol­ecular building blocks. Much attention has been devoted to the cogitative design and control of self-assembly of infinite coordination networks by careful selection of ligand geometry (Liu *et al.*, 2016 ▸; Yoon *et al.*, 2012 ▸). In this regard, the use of symmetrical ligands has been a successful paradigm because of their structural predictability (Rosi *et al.*, 2003 ▸; Luo *et al.*, 2003 ▸). Incorporation of unsymmetrical ligands in such systems, however, is relatively recent (Wang *et al.*, 2004 ▸; Chen *et al.*, 2003 ▸; Qin *et al.*, 2005 ▸). Compared to symmetrical ligands, ligands with two or more coordination sites with differing donor ability can lead to unsymmetrical ligands being assembled around metal atoms in diverse arrangements. This can result in unprecedented structures with novel topological features, such as a clay-like double layer (Pan *et al.*, 2000 ▸), large spherical cavities and functional 1D channels (Shin *et al.*, 2003 ▸). Although important progress has been made in the construction of coordination polymers by applying a single type of organic ligand, research involving a combination of more than one ligand is an especially attractive target, as it allows the construction of an almost infinite number of frameworks with different crystal structures.

In our work, we use (*E*)-3-[3-(carb­oxy­meth­oxy)phen­yl]acrylic acid (H_2_ *L*) and 3-(pyridin-4-yl)pyrazole (pp) as ligands to construct novel MOFs that are based on the following considerations: (1) the carboxyl­ate group is conjugated with the benzene ring through a C=C double bond, which makes the electron density delocalized in the ligand so that it may become more rigid when coordinating to metal ions, and have more coordination modes and conformation changes (Kong *et al.*, 2013 ▸; Liu *et al.*, 2010 ▸); (2) the presence of a phenolic hydroxyl group and benzene ring in the ligand allows the possibility of hydrogen bonding and π–π stacking inter­actions in the crystal lattices; (3) the N-donor ligand could enhance structural stability.

We herein report the synthesis and crystal structure of [Co(C_11_H_8_O_5_)(C_8_H_7_N_3_)]_*n*_ based on these two mixed ligands.
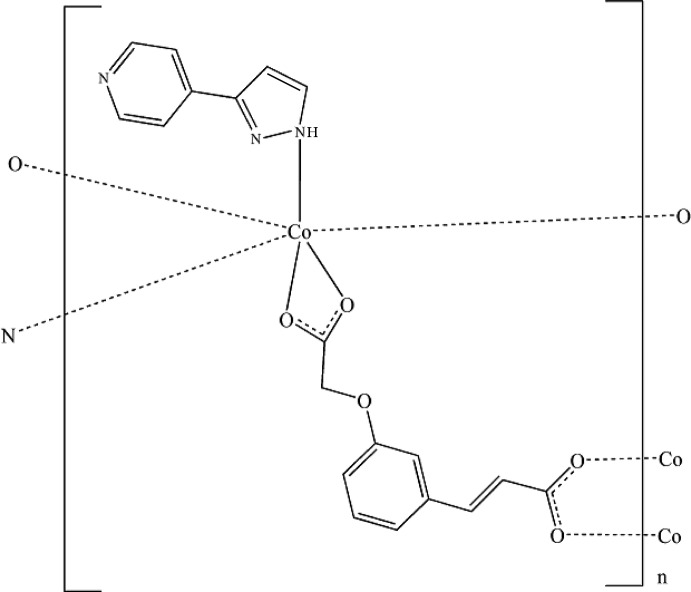


## Structural commentary   

**Figure 1:**
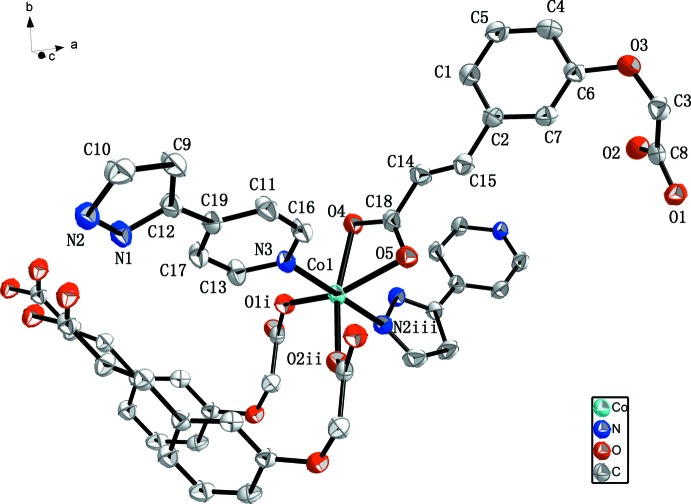
The coordination environment of the Co^2+^ ion in the title complex (omitting all H atoms), showing the atom-numbering scheme for non-H atoms. Displacement ellipsoids are drawn at the 40% probability level. [Symmetry codes: (i) *x*, *y*, *z* − 1; (ii) *x* − 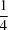, 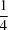 − *y*, *z* + 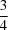; (iii) *x* + 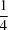, 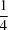 − *y*, *z* + 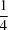.]

**Figure 2:**
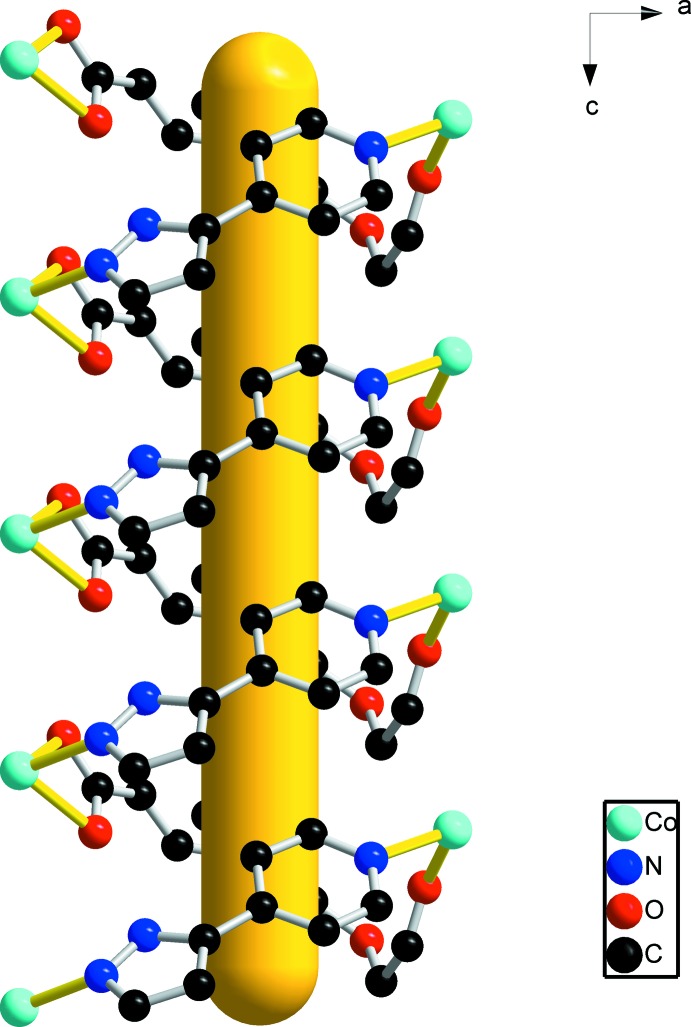
The helical chain in the title compound (omitting all H atoms). The yellow rod indicates the direction of propagation of the helix (*i.e.* parallel to the *c* axis).

**Figure 3:**
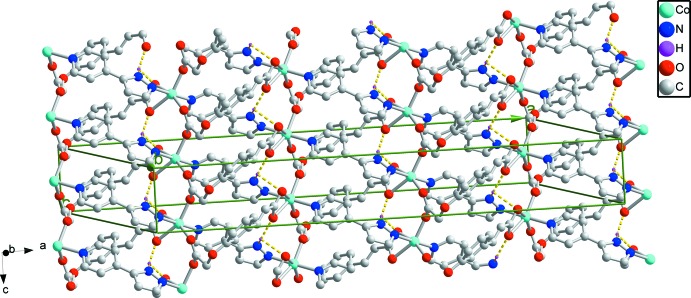
The two-dimensional packing of the title compound. Hydrogen bonds are depicted as dashed lines.

As shown in Fig. 1 ▸, the asymmetric unit of the title compound comprises one Co^2+^ cation, one fully deprotonated *L* ^2−^ anion, and one pp ligand. The Co^II^ atom has a distorted octa­hedral geometry, coordinated by four O atoms from three *L* ^2−^ ligands, with Co^II^—O distances of 2.037 (2)–2.252 (2) Å, and two N atoms from two pp ligands with Co^II^—N distances of 2.130 (2) and 2.158 (3) Å. The *L* ^2−^ ligand adopts two different coordination modes. In this structure, the dihedral angles between the rings in the pp ligands is 23.1 (2)°. The 1D helical chains (Fig. 2 ▸) are assembled by Co^2+^ cations, pp ligands and *L* ligands. Helical chains along the *c* axis are connected to adjacent chains by *L* ligands that bridge the Co^II^ atoms, forming a two-dimensional polymeric structure in the *ac* plane (Fig. 3 ▸).

In the structure, every η^3^-(*E*)-3-[3-(carb­oxy­meth­oxy)phen­yl]acrylic acid ligand is connected to three Co atoms, while every η^3^-3-(pyridin-4-yl)pyrazole is connected to two Co atoms. The Co^II^ atom connects three *L* ^2−^ ligands and two pp ligands, and so can be described as a five-connected node. Thus, the topology of the structure could be given simply as a (2,3,5)-connected network.

## Supra­molecular features   

In this structure, *L* ligands form hydrogen bonds to the pp ligands, thereby enhancing the polymer stability (Table 1 ▸ and Fig. 3 ▸). The polymer inter­actions consist of N1(pyrazole)—H1*A*⋯O5(*x* − 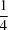, −*y* + 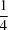, *z* − 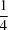) hydrogen bonds where each *L* ligand makes a hydrogen bond with a neighboring pp ligand.

## Database survey   

The crystal structure of a 2D polymeric Cd-containing compound with (*E*)-3-(3-carb­oxy­meth­oxy)phen­yl)acrylic acid and 1,3-di-pyridin-4-yl­propane ligands (the Cd-crystal), recently reported by Wang *et al.* (2014 ▸), has a similar structure to the title compound. Both structures include hydrogen bonds, though in the Cd-crystal, these are O—H⋯O hydrogen bonds rather than N—H⋯O as in the title compound.

## Synthesis and crystallization   

All of the chemical reagents and solvents are commercially available and used without further purification. Elemental analyses were carried out on a Perkin–Elmer 2400 Series II analyzer.

**Synthesis of [Co(C_11_H_8_O_5_)(C_8_H_7_N_3_)]** ***_n_***

(1): A mixture of CoCl_2_·6H_2_O (0.1185 g, 0.5 mmol), H_2_ *L* (Zheng *et al.*, 2011 ▸; Fu & Wen, 2011 ▸) (0.222 g, 1 mmol) and pp (0.1451 g, 1 mmol) were dissolved in 22 mL H_2_O/CH_3_OH (*v*/*v*, 10:1) mixed solvent. The pH value was adjusted to 7 by adding to a few drops of an aqueous NaOH solution (2.0 mol L^−1^). It was then sealed in a 25 mL stainless steel reactor and heated to 433 K for three days. The mixture was then cooled to room temperature at a rate of 5 K h^−1^, and red block-shaped crystals were obtained (yield: 62% based on Co). Analysis calculated (%) for C_19_H_15_CoN_3_O_5_ (424.27): C 53.81, H 3.62, N 9.85; found (%): C 53.79, H 3.56, N 9.90. IR data (KBr, cm^−1^): 3432, 1649, 1501, 1407, 1274, 1206, 1180, 1086, 978, 844, 724, 603.

## Refinement   

Crystal data, data collection and structure refinement details are summarized in Table 2 ▸. Hydrogen atoms attached to carbon atoms were refined using a riding-model approximation, with *U*_iso_(H) = 1.2*U*_eq_(C) and C—H = 0.93 Å (aromatic and carbene) and 0.97 Å (methyl­ene). Other hydrogen atoms were located in difference electron-density maps and refined freely.

## Supplementary Material

Crystal structure: contains datablock(s) I. DOI: 10.1107/S205698901601402X/pk2590sup1.cif

Structure factors: contains datablock(s) I. DOI: 10.1107/S205698901601402X/pk2590Isup3.hkl

CCDC reference: 1502210

Additional supporting information: crystallographic information; 3D view; checkCIF report

## Figures and Tables

**Table 1:** Hydrogen-bond geometry (Å, °)

*D*—H⋯*A*	*D*—H	H⋯*A*	*D*⋯*A*	*D*—H⋯*A*
N1—H1*A*⋯O5^i^	0.86	2.05	2.869 (3)	159

Symmetry code: (i) 
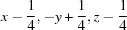
.

**Table 2:** Experimental details

Crystal data	
Chemical formula	[Co(C_11_H_8_O_5_)(C_8_H_7_N_3_)]
*M*_r_	424.27
Crystal system, space group	Orthorhombic, *F* *d* *d*2
Temperature (K)	296
*a*, *b*, *c* (Å)	35.4631 (11), 40.2873 (12), 4.8423 (1)
*V* (Å^3^)	6918.3 (3)
*Z*	16
Radiation type	Mo *K*α
μ (mm^−1^)	1.03
Crystal size (mm)	0.24 × 0.12 × 0.06

Data collection	
Diffractometer	Bruker APEXII CCD
Absorption correction	Multi-scan (*SADABS*; Bruker, 2014 ▸)
*T*_min_, *T*_max_	0.861, 0.943
No. of measured, independent and observed [*I* > 2σ(*I*)] reflections	15248, 3915, 3425
*R*_int_	0.036
(sin θ/λ)_max_ (Å^−1^)	0.651

Refinement	
*R*[*F* ^2^ > 2σ(*F* ^2^)], *wR*(*F* ^2^), *S*	0.029, 0.061, 1.02
No. of reflections	3915
No. of parameters	253
No. of restraints	1
H-atom treatment	H-atom parameters constrained
Δρ_max_, Δρ_min_ (e Å^−3^)	0.18, −0.24
Absolute structure	Flack *x* determined using 1316 quotients [(*I* ^+^)−(*I* ^−^)]/[(*I* ^+^)+(*I* ^−^)] (Parsons *et al.*, 2013 ▸)
Absolute structure parameter	0.025 (8)

Computer programs: *APEX2* and *SAINT-Plus* (Bruker, 2014 ▸), *SHELXS97* and *SHELXTL* (Sheldrick, 2008 ▸), *SHELXL2014* (Sheldrick, 2015 ▸) and *publCIF* (Westrip, 2010 ▸).

## supplementary crystallographic information

### Crystal data

**Table:**

[Co(C_11_H_8_O_5_)(C_8_H_7_N_3_)]	*D*_x_ = 1.629 Mg m^−^^3^
*M**_r_* = 424.27	Mo *K*α radiation, λ = 0.71073 Å
Orthorhombic, *F**d**d*2	Cell parameters from 4631 reflections
*a* = 35.4631 (11) Å	θ = 1.5–27.6°
*b* = 40.2873 (12) Å	µ = 1.03 mm^−^^1^
*c* = 4.8423 (1) Å	*T* = 296 K
*V* = 6918.3 (3) Å^3^	Block, red
*Z* = 16	0.24 × 0.12 × 0.06 mm
*F*(000) = 3472	

### Data collection

**Table:**

Bruker APEXII CCD diffractometer	3425 reflections with *I* > 2σ(*I*)
ω and φ scans	*R*_int_ = 0.036
Absorption correction: multi-scan (SADABS; Bruker, 2014)	θ_max_ = 27.6°, θ_min_ = 1.5°
*T*_min_ = 0.861, *T*_max_ = 0.943	*h* = −46→40
15248 measured reflections	*k* = −48→52
3915 independent reflections	*l* = −6→6

### Refinement

**Table:**

Refinement on *F*^2^	Secondary atom site location: difference Fourier map
Least-squares matrix: full	Hydrogen site location: inferred from neighbouring sites
*R*[*F*^2^ > 2σ(*F*^2^)] = 0.029	H-atom parameters constrained
*wR*(*F*^2^) = 0.061	*w* = 1/[σ^2^(*F*_o_^2^) + (0.0282*P*)^2^] where *P* = (*F*_o_^2^ + 2*F*_c_^2^)/3
*S* = 1.02	(Δ/σ)_max_ = 0.001
3915 reflections	Δρ_max_ = 0.18 e Å^−^^3^
253 parameters	Δρ_min_ = −0.24 e Å^−^^3^
1 restraint	Absolute structure: Flack *x* determined using 1316 quotients [(*I*^+^)-(*I*^-^)]/[(*I*^+^)+(*I*^-^)] (Parsons *et al.*, 2013)
Primary atom site location: structure-invariant direct methods	Absolute structure parameter: 0.025 (8)

### Special details

**Table:**

Geometry. All esds (except the esd in the dihedral angle between two l.s. planes) are estimated using the full covariance matrix. The cell esds are taken into account individually in the estimation of esds in distances, angles and torsion angles; correlations between esds in cell parameters are only used when they are defined by crystal symmetry. An approximate (isotropic) treatment of cell esds is used for estimating esds involving l.s. planes.

### Fractional atomic coordinates and isotropic or equivalent isotropic displacement parameters (Å^2^)

**Table:**

	*x*	*y*	*z*	*U*_iso_*/*U*_eq_	
Co1	0.43282 (2)	0.10099 (2)	0.42130 (8)	0.02757 (11)	
N1	0.25519 (6)	0.16432 (6)	0.8665 (5)	0.0315 (6)	
H1A	0.2498	0.1481	0.7576	0.038*	
N2	0.23011 (7)	0.17869 (6)	1.0382 (6)	0.0344 (6)	
N3	0.38578 (7)	0.13040 (6)	0.5432 (5)	0.0302 (6)	
O1	0.65789 (6)	0.15999 (5)	1.3036 (4)	0.0351 (5)	
O2	0.66654 (5)	0.18826 (5)	0.9144 (4)	0.0334 (5)	
O3	0.63324 (6)	0.24391 (5)	1.1390 (5)	0.0379 (5)	
O4	0.45839 (6)	0.14604 (5)	0.2794 (4)	0.0361 (5)	
O5	0.47664 (6)	0.12681 (5)	0.6803 (5)	0.0359 (5)	
C1	0.54127 (9)	0.24388 (8)	0.6113 (6)	0.0388 (8)	
H1B	0.5214	0.2445	0.4858	0.047*	
C2	0.54793 (8)	0.21527 (7)	0.7657 (6)	0.0317 (7)	
C3	0.64159 (9)	0.21627 (7)	1.3104 (6)	0.0353 (7)	
H3A	0.6188	0.2100	1.4085	0.042*	
H3B	0.6600	0.2231	1.4470	0.042*	
C4	0.59389 (9)	0.27054 (8)	0.8261 (7)	0.0376 (8)	
H4A	0.6088	0.2893	0.8492	0.045*	
C5	0.56410 (9)	0.27116 (8)	0.6447 (8)	0.0418 (8)	
H5A	0.5593	0.2903	0.5432	0.050*	
C6	0.60175 (8)	0.24195 (7)	0.9746 (6)	0.0307 (7)	
C7	0.57842 (8)	0.21459 (7)	0.9493 (7)	0.0319 (7)	
H7A	0.5830	0.1957	1.0545	0.038*	
C8	0.65663 (7)	0.18596 (7)	1.1621 (7)	0.0280 (6)	
C9	0.28655 (9)	0.20335 (8)	1.0763 (8)	0.0433 (9)	
H9A	0.3054	0.2178	1.1346	0.052*	
C10	0.24921 (9)	0.20257 (8)	1.1639 (8)	0.0421 (8)	
H10A	0.2390	0.2170	1.2940	0.050*	
C11	0.35849 (9)	0.17039 (8)	0.8458 (7)	0.0374 (8)	
H11A	0.3621	0.1865	0.9809	0.045*	
C12	0.28963 (8)	0.17826 (7)	0.8854 (7)	0.0319 (7)	
C13	0.35135 (8)	0.12506 (8)	0.4394 (7)	0.0362 (7)	
H13A	0.3488	0.1099	0.2961	0.043*	
C14	0.50252 (9)	0.17858 (8)	0.5230 (7)	0.0370 (7)	
H14A	0.5028	0.1935	0.3763	0.044*	
C15	0.52392 (8)	0.18555 (8)	0.7372 (6)	0.0342 (8)	
H15A	0.5239	0.1705	0.8827	0.041*	
C16	0.38898 (9)	0.15306 (7)	0.7424 (6)	0.0352 (8)	
H16A	0.4128	0.1574	0.8150	0.042*	
C17	0.31952 (9)	0.14106 (8)	0.5349 (7)	0.0363 (8)	
H17A	0.2961	0.1366	0.4568	0.044*	
C18	0.47794 (8)	0.14901 (8)	0.4955 (6)	0.0313 (7)	
C19	0.32276 (8)	0.16387 (8)	0.7491 (6)	0.0309 (7)	

### Atomic displacement parameters (Å^2^)

**Table:**

	*U*^11^	*U*^22^	*U*^33^	*U*^12^	*U*^13^	*U*^23^
Co1	0.02385 (19)	0.0318 (2)	0.02701 (18)	−0.00149 (18)	0.00226 (17)	0.00073 (18)
N1	0.0247 (13)	0.0366 (15)	0.0333 (15)	−0.0018 (11)	0.0050 (10)	−0.0072 (11)
N2	0.0285 (14)	0.0358 (15)	0.0387 (14)	0.0004 (12)	0.0066 (12)	−0.0056 (12)
N3	0.0253 (14)	0.0347 (14)	0.0306 (12)	−0.0022 (12)	0.0030 (11)	0.0001 (11)
O1	0.0423 (13)	0.0337 (12)	0.0295 (10)	0.0020 (10)	−0.0026 (10)	0.0017 (10)
O2	0.0352 (11)	0.0390 (12)	0.0260 (10)	0.0035 (9)	0.0074 (10)	−0.0010 (10)
O3	0.0402 (12)	0.0299 (11)	0.0436 (13)	−0.0026 (9)	−0.0066 (11)	−0.0044 (10)
O4	0.0354 (13)	0.0376 (13)	0.0353 (11)	−0.0051 (10)	−0.0053 (10)	0.0027 (10)
O5	0.0340 (12)	0.0394 (12)	0.0344 (11)	−0.0054 (9)	0.0011 (10)	0.0060 (11)
C1	0.0398 (19)	0.0368 (19)	0.040 (2)	0.0030 (15)	−0.0050 (14)	0.0001 (14)
C2	0.0291 (17)	0.0310 (17)	0.0350 (16)	0.0008 (14)	0.0033 (12)	−0.0033 (13)
C3	0.0378 (18)	0.0386 (19)	0.0296 (15)	0.0037 (15)	−0.0030 (14)	−0.0058 (14)
C4	0.044 (2)	0.0277 (17)	0.0409 (19)	−0.0048 (15)	0.0034 (15)	−0.0002 (13)
C5	0.051 (2)	0.0308 (17)	0.0437 (19)	0.0012 (15)	−0.0009 (17)	0.0048 (16)
C6	0.0306 (17)	0.0311 (16)	0.0304 (17)	0.0005 (14)	0.0024 (11)	−0.0074 (12)
C7	0.0356 (17)	0.0272 (15)	0.0328 (16)	0.0013 (12)	0.0008 (14)	−0.0012 (13)
C8	0.0219 (14)	0.0351 (16)	0.0268 (14)	−0.0016 (12)	−0.0028 (13)	−0.0022 (14)
C9	0.0301 (19)	0.0379 (19)	0.062 (2)	−0.0087 (15)	0.0071 (16)	−0.0138 (16)
C10	0.0426 (19)	0.0348 (17)	0.0488 (18)	0.0030 (15)	0.0095 (18)	−0.0110 (18)
C11	0.0329 (18)	0.0360 (18)	0.043 (2)	−0.0054 (15)	0.0019 (13)	−0.0144 (14)
C12	0.0243 (16)	0.0316 (16)	0.0399 (18)	−0.0013 (13)	0.0045 (13)	−0.0033 (14)
C13	0.0281 (17)	0.0462 (18)	0.0343 (16)	−0.0019 (14)	−0.0006 (14)	−0.0098 (16)
C14	0.0370 (18)	0.0350 (18)	0.0388 (17)	−0.0061 (15)	0.0014 (14)	0.0035 (14)
C15	0.0313 (17)	0.0331 (18)	0.0383 (19)	0.0017 (14)	0.0015 (13)	−0.0001 (13)
C16	0.0264 (17)	0.0401 (19)	0.039 (2)	−0.0046 (14)	0.0011 (12)	−0.0058 (13)
C17	0.0238 (17)	0.043 (2)	0.0422 (17)	0.0014 (14)	−0.0007 (13)	−0.0082 (16)
C18	0.0239 (16)	0.0350 (18)	0.0351 (18)	0.0007 (14)	0.0046 (12)	−0.0021 (13)
C19	0.0251 (16)	0.0315 (17)	0.0362 (18)	−0.0001 (14)	0.0040 (11)	0.0005 (13)

### Geometric parameters (Å, º)

**Table:**

Co1—O1^i^	2.037 (2)	C3—C8	1.514 (4)
Co1—O2^ii^	2.054 (2)	C3—H3A	0.9700
Co1—N3	2.130 (2)	C3—H3B	0.9700
Co1—O4	2.142 (2)	C4—C5	1.374 (4)
Co1—N2^iii^	2.158 (3)	C4—C6	1.386 (4)
Co1—O5	2.252 (2)	C4—H4A	0.9300
N1—C12	1.347 (4)	C5—H5A	0.9300
N1—N2	1.348 (3)	C6—C7	1.384 (4)
N1—H1A	0.8600	C7—H7A	0.9300
N2—C10	1.325 (4)	C9—C12	1.374 (4)
N2—Co1^iv^	2.158 (3)	C9—C10	1.391 (4)
N3—C16	1.333 (4)	C9—H9A	0.9300
N3—C13	1.338 (4)	C10—H10A	0.9300
O1—C8	1.251 (3)	C11—C19	1.376 (4)
O1—Co1^v^	2.037 (2)	C11—C16	1.381 (4)
O2—C8	1.253 (4)	C11—H11A	0.9300
O2—Co1^vi^	2.054 (2)	C12—C19	1.467 (4)
O3—C6	1.374 (3)	C13—C17	1.380 (4)
O3—C3	1.420 (3)	C13—H13A	0.9300
O4—C18	1.261 (4)	C14—C15	1.316 (4)
O5—C18	1.266 (3)	C14—C18	1.482 (4)
C1—C5	1.375 (4)	C14—H14A	0.9300
C1—C2	1.394 (4)	C15—H15A	0.9300
C1—H1B	0.9300	C16—H16A	0.9300
C2—C7	1.400 (4)	C17—C19	1.390 (4)
C2—C15	1.476 (4)	C17—H17A	0.9300
			
O1^i^—Co1—O2^ii^	102.24 (8)	C4—C5—H5A	119.5
O1^i^—Co1—N3	91.33 (9)	C1—C5—H5A	119.5
O2^ii^—Co1—N3	92.82 (9)	O3—C6—C7	125.7 (3)
O1^i^—Co1—O4	95.01 (8)	O3—C6—C4	114.6 (3)
O2^ii^—Co1—O4	162.75 (8)	C7—C6—C4	119.7 (3)
N3—Co1—O4	87.08 (9)	C6—C7—C2	120.1 (3)
O1^i^—Co1—N2^iii^	87.48 (10)	C6—C7—H7A	119.9
O2^ii^—Co1—N2^iii^	87.89 (9)	C2—C7—H7A	119.9
N3—Co1—N2^iii^	178.72 (11)	O1—C8—O2	125.2 (3)
O4—Co1—N2^iii^	92.56 (9)	O1—C8—C3	115.3 (3)
O1^i^—Co1—O5	152.48 (8)	O2—C8—C3	119.5 (3)
O2^ii^—Co1—O5	103.33 (8)	C12—C9—C10	105.3 (3)
N3—Co1—O5	97.42 (9)	C12—C9—H9A	127.3
O4—Co1—O5	59.66 (8)	C10—C9—H9A	127.3
N2^iii^—Co1—O5	83.45 (9)	N2—C10—C9	111.3 (3)
C12—N1—N2	112.2 (2)	N2—C10—H10A	124.4
C12—N1—H1A	123.9	C9—C10—H10A	124.4
N2—N1—H1A	123.9	C19—C11—C16	120.1 (3)
C10—N2—N1	104.9 (2)	C19—C11—H11A	120.0
C10—N2—Co1^iv^	131.5 (2)	C16—C11—H11A	120.0
N1—N2—Co1^iv^	117.32 (19)	N1—C12—C9	106.3 (3)
C16—N3—C13	117.4 (3)	N1—C12—C19	122.1 (3)
C16—N3—Co1	121.0 (2)	C9—C12—C19	131.1 (3)
C13—N3—Co1	121.4 (2)	N3—C13—C17	123.1 (3)
C8—O1—Co1^v^	132.5 (2)	N3—C13—H13A	118.5
C8—O2—Co1^vi^	124.8 (2)	C17—C13—H13A	118.5
C6—O3—C3	117.6 (2)	C15—C14—C18	125.6 (3)
C18—O4—Co1	92.70 (18)	C15—C14—H14A	117.2
C18—O5—Co1	87.59 (18)	C18—C14—H14A	117.2
C5—C1—C2	119.9 (3)	C14—C15—C2	125.4 (3)
C5—C1—H1B	120.1	C14—C15—H15A	117.3
C2—C1—H1B	120.1	C2—C15—H15A	117.3
C1—C2—C7	119.2 (3)	N3—C16—C11	122.8 (3)
C1—C2—C15	121.5 (3)	N3—C16—H16A	118.6
C7—C2—C15	119.3 (3)	C11—C16—H16A	118.6
O3—C3—C8	115.4 (3)	C13—C17—C19	119.4 (3)
O3—C3—H3A	108.4	C13—C17—H17A	120.3
C8—C3—H3A	108.4	C19—C17—H17A	120.3
O3—C3—H3B	108.4	O4—C18—O5	120.0 (3)
C8—C3—H3B	108.4	O4—C18—C14	118.3 (3)
H3A—C3—H3B	107.5	O5—C18—C14	121.7 (3)
C5—C4—C6	120.1 (3)	C11—C19—C17	117.1 (3)
C5—C4—H4A	120.0	C11—C19—C12	120.6 (3)
C6—C4—H4A	120.0	C17—C19—C12	122.0 (3)
C4—C5—C1	120.9 (3)		
			
C12—N1—N2—C10	1.1 (3)	C10—C9—C12—N1	0.5 (4)
C12—N1—N2—Co1^iv^	−154.4 (2)	C10—C9—C12—C19	−170.5 (3)
C5—C1—C2—C7	−1.3 (5)	C16—N3—C13—C17	−2.1 (5)
C5—C1—C2—C15	179.6 (3)	Co1—N3—C13—C17	172.6 (3)
C6—O3—C3—C8	73.1 (3)	C18—C14—C15—C2	−179.0 (3)
C6—C4—C5—C1	1.2 (5)	C1—C2—C15—C14	21.2 (5)
C2—C1—C5—C4	0.9 (5)	C7—C2—C15—C14	−157.9 (3)
C3—O3—C6—C7	−3.8 (4)	C13—N3—C16—C11	1.1 (4)
C3—O3—C6—C4	176.1 (3)	Co1—N3—C16—C11	−173.7 (2)
C5—C4—C6—O3	177.0 (3)	C19—C11—C16—N3	2.0 (5)
C5—C4—C6—C7	−3.1 (5)	N3—C13—C17—C19	0.0 (5)
O3—C6—C7—C2	−177.4 (3)	Co1—O4—C18—O5	3.0 (3)
C4—C6—C7—C2	2.7 (4)	Co1—O4—C18—C14	−178.0 (2)
C1—C2—C7—C6	−0.5 (4)	Co1—O5—C18—O4	−2.8 (3)
C15—C2—C7—C6	178.6 (3)	Co1—O5—C18—C14	178.2 (3)
Co1^v^—O1—C8—O2	124.6 (3)	C15—C14—C18—O4	178.6 (3)
Co1^v^—O1—C8—C3	−55.7 (3)	C15—C14—C18—O5	−2.4 (5)
Co1^vi^—O2—C8—O1	5.7 (4)	C16—C11—C19—C17	−4.0 (5)
Co1^vi^—O2—C8—C3	−174.00 (19)	C16—C11—C19—C12	170.6 (3)
O3—C3—C8—O1	−168.3 (2)	C13—C17—C19—C11	3.0 (5)
O3—C3—C8—O2	11.4 (4)	C13—C17—C19—C12	−171.4 (3)
N1—N2—C10—C9	−0.7 (4)	N1—C12—C19—C11	−156.1 (3)
Co1^iv^—N2—C10—C9	149.7 (3)	C9—C12—C19—C11	13.7 (5)
C12—C9—C10—N2	0.2 (4)	N1—C12—C19—C17	18.2 (5)
N2—N1—C12—C9	−1.0 (4)	C9—C12—C19—C17	−172.0 (3)
N2—N1—C12—C19	171.0 (3)		

Symmetry codes: (i) *x*−1/4, −*y*+1/4, *z*−5/4; (ii) *x*−1/4, −*y*+1/4, *z*−1/4; (iii) *x*+1/4, −*y*+1/4, *z*−3/4; (iv) *x*−1/4, −*y*+1/4, *z*+3/4; (v) *x*+1/4, −*y*+1/4, *z*+5/4; (vi) *x*+1/4, −*y*+1/4, *z*+1/4.

### Hydrogen-bond geometry (Å, º)

**Table:**

*D*—H···*A*	*D*—H	H···*A*	*D*···*A*	*D*—H···*A*
N1—H1*A*···O5^ii^	0.86	2.05	2.869 (3)	159

Symmetry code: (ii) *x*−1/4, −*y*+1/4, *z*−1/4.
